# Molecular Conformations of Di-, Tri-, and Tetra-*α*-(2→8)-Linked Sialic Acid from NMR Spectroscopy and MD Simulations

**DOI:** 10.3390/ijms21010030

**Published:** 2019-12-19

**Authors:** Aysegül Turupcu, Markus Blaukopf, Paul Kosma, Chris Oostenbrink

**Affiliations:** 1 Department of Material Sciences and Process Engineering, Institute of Molecular Modeling and Simulation, University of Natural Resources and Life Sciences Vienna, 1190 Vienna, Austria; aturupcu@boku.ac.at; 2 Department of Chemistry, University of Natural Resources and Life Sciences Vienna, 1190 Vienna, Austria; markus.blaukopf@boku.ac.at (M.B.); paul.kosma@boku.ac.at (P.K.)

**Keywords:** sialic acid, conformational analysis, molecular dynamics, nuclear magnetic resonance

## Abstract

By using molecular dynamics simulations with an efficient enhanced sampling technique and in combination with nuclear magnetic resonance (NMR) spectroscopy quantitative structural information on α-2,8-linked sialic acids is presented. We used a bottom-up approach to obtain a set of larger ensembles for tetra- and deca-sialic acid from model dimer and trimer systems that are in agreement with the available J-coupling constants and nuclear Overhauser effects. The molecular dynamic (MD) simulations with enhanced sampling are used to validate the force field used in this study for its further use. This empowered us to couple NMR observables in the MD framework via J-coupling and distance restraining simulations to obtain conformations that are supported by experimental data. We used these conformations in thermodynamic integration and one-step perturbation simulations to calculate the free-energy of suggested helical conformations. This study brings most of the available NMR experiments together and supplies information to resolve the conflict on the structures of poly-α-2,8-linked sialic acid.

## 1. Introduction

Neuraminic (sialic) acids are commonly found in the carbohydrate moiety of glycoproteins and glycolipids, typically at the outermost non-reducing ends. The chemical nature of this sugar, being negatively charged and hydrophilic, makes it suitable to perform key roles in molecular recognition events. In addition, sialic acids can form polymers with degrees of polymerization varying from 8 to 200, called polysialic acids (polySia) [1].

Polysialic acids play crucial roles in various steps of neural development, such as cell survival and cell migration of neural precursors, neuronal guidance, and synapse formation [2]. Modification of therapeutic recombinant proteins by polySia is of growing pharmaceutical relevance due to the significant increase in their serum lifetime [3,4]. However, the structural properties of these polymeric units are still under debate. First, Jennings et al. [5] found that at least a decamer of N-acetylated neuraminic acid (Neu5Ac)${}_{10}$ was needed for binding to the group B polysaccharide specific antibody. As the size of the antibody is limited to the accommodation of 6–7 residues, α-2,8-linked polySia was considered to form a structural epitope. After that, structural studies on the conformation of oligomers of different degrees of polymerization suggested a flexible helical structure by early NMR [6,7]. One model was built from ${}^{3}$J${}_{HH}$ coupling constants by setting the appropriate torsional angles and using the antibody binding site as a target. This resulted in a helical model with 6 residues per turn [8], contradicting another helical polymer model, which was built by setting torsional angles satisfying coupling constants and suggests 3–4 residues per turn [9]. Yongye et al. [10] suggested another helical type by taking trisialic acid as a model through a combination of NMR and MD studies. Battistel et al. observed transient H-bonds in α-2,8-linked tetra-sialic acid from ${}^{3}$J${}_{N-C}$ correlations at 263 K [11]. A contrasting NMR study based on ${}^{13}$C-{${}^{1}$H} NMR relaxation times and steady-state NOE’s suggests a random coil rather than a helical structure [12]. The actual conformation(s) are still unclear since there is no consensus on either the type of the helical conformation or on the random coil.

In the current work, we perform molecular simulations of di-, tri-, and tetra- α-2,8-linked sialic acid to study its conformational freedom and preferences. The simulations will be compared to and biased by experimental data derived from NMR experiments which were both conducted in this study and available in the literature, to characterize the conformational flexibility, within the limits set by the measured data. This study allowed us to further comment on the conformation on polymeric sialic acid with a decamer model not by using indirect information through setting torsional angles from coupling constants but by direct use of NOEs and coupling constants in the simulation leading to an ensemble satisfying the experimental findings.

**Figure 1:**
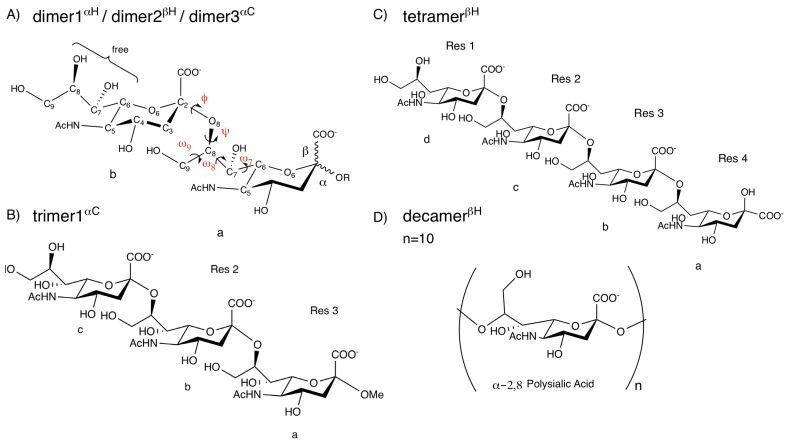
Studied systems. (**A**) Schematic representation of dimer systems R=H or C, with the anomericity indicated in the superscript (**B**); trimer with residue labels (**C**); tetramer with residue labels; (**D**) decamer with n=10. Definition of the dihedral angles are ϕ =O6-C2-O8’-C8’, ψ=C2-O8’-C8’-C7’, ω7=O7-C7-C6-O6, ω8=O8-C8-C7-O7, and ω9=O9-C9-C8-O8.

Initially, we have studied the conformational landscapes of α-2,8-linked sialic acids in three different dimers, one trimer, and one tetramer (see Figure 1). The analyzed dimers were subsequently used to model larger sialic acid systems from tri-, tetra- to decasialic acid. In contrast to the α-linkages in polysialic acid, the reducing end, residue a, is present as the β-anomer which has a major impact on the NMR-characteristics of this unit and as well as of the preceding residue b. Therefore, for building tetra-sialic acid, we have used the information from its exact disaccharide units with respective anomericity. In the trimer system, the reducing end was kept in α-anomeric form to study the effect of chain prolongation on the dimeric linkage.

## 2. Results and Discussion

α-2,8-linked polySia has gained interest in recent drug design, especially in the development of specific antibodies and for glycoengineering of therapeutic proteins. However, the conformation of this polymer remains unsolved because of incomplete information on its structure. To shed light on the structure of α-2,8-linked tetra-sialic acid and the conformational preferences of its dimeric units we have performed various molecular simulations and validated these against NMR experiments. We previously used this approach to obtain 3D structures of various glycan units where the structure was unknown [13,14]. The 53A6${}_{GLYC}$ [15] force field was used both to validate the sialic acid conformation and use it to propose conformational preferences of higher units. This force field was shown to reproduce experimental observables (NOE’s, ${}^{3}$J coupling constants, etc.) well [16,17].

We have already shown in our recent work [17] that the LEUS simulations offer a more complete sampling than unbiased MD simulations. Therefore, we chose to compare the NMR findings with the LEUS simulations. For the three dimers, one trimer, and one tetramer, LEUS simulations were analyzed in terms of ${}^{3}$J${}_{H8H9R/S}$, ${}^{3}$J${}_{H7H8}$, and ${}^{3}$J${}_{H6H7}$ coupling constants, intramolecular hydrogen bonding occurrences, and glycosidic dihedral angle distributions. All ensemble averages were unbiased using Equation (1) (see Section 3).

### 2.1. Free-Energy Landscape

**Figure 2:**
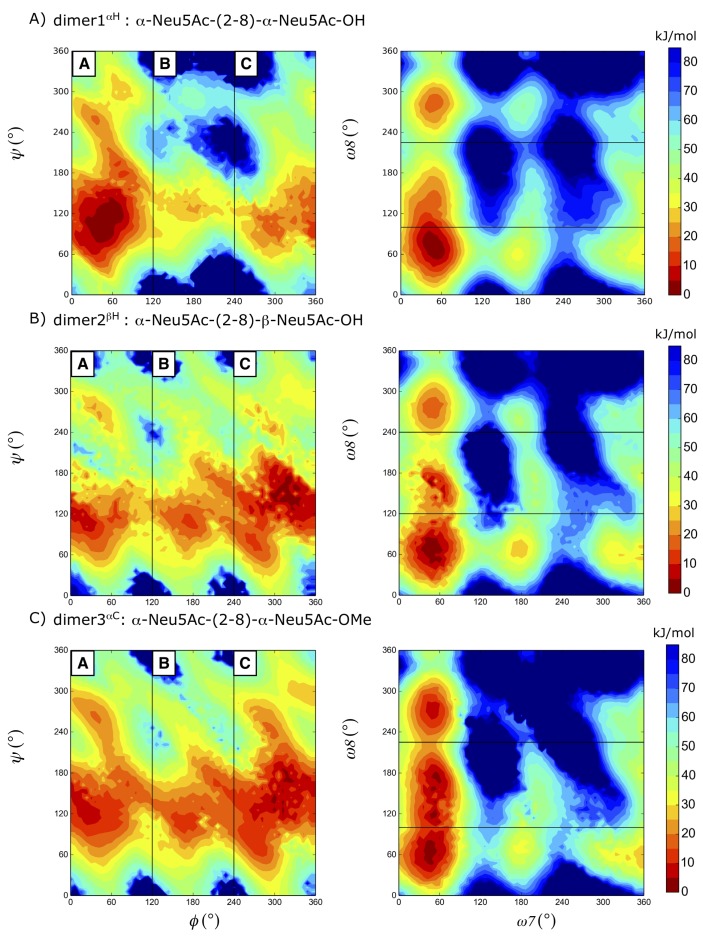
Free-energy maps G (ϕ, ψ) and G (ω7, ω8) of the glycosidic dihedral angles from the local elevation of umbrella sampling (LEUS) simulations. Contour maps are drawn with 5 kJ/mol spacing starting from the global minimum energy which is set to 0 kJ/mol. The regions that were never visited are shown in dark blue and the corresponding unbiased free energies are represented in the color maps. (**A**) dimer1^αH^; (**B**) dimer2^βH^ (**C**); dimer3^αC^.

Inspection of the glycosidic free-energy maps (Figure 2) reveals that the α-2,8-linked systems show three significant minima regions (termed as A, B, and C) unlike other 1→n (where *n* is 2, 3, 4, and 6) linked disaccharides displaying four regions (see Ref. [18]). Although this behavior was shared among the studied dimer systems, the population of the states differs, depending on the stereochemistry of the reducing end. The corresponding states (A, B, C) have free-energy values of 0.0, 23.3, 13.67 kJ mol${}^{-1}$ for dimer1^αH^ and 6.6, 12.1, 0.0 kJ mol${}^{-1}$ for dimer2^βH^, respectively. In region A, the minimum for the ϕ dihedral angle slightly shifts towards 30${}^{\circ }$ in dimer2 and dimer3 compared to dimer1, for which it is centered around 60${}^{\circ }$ (for ψ = 120${}^{\circ }$). The additional degree of freedom, ω8 in these linkages is almost freely rotating while ω7 is restricted to 60${}^{\circ }$ as can be seen in Figure 2. This predominance of the g${}^{+}$ conformation of ω7 is in agreement with NMR studies [6,7]. Although the right upper part of the ϕ,ψ free-energy landscape became more favorable compared to dimer1^αH^, as expected, the effect of the end-group in dimer3^αC^ is relatively minor.

### 2.2. ^3^J Coupling Constants

For systems dimer1^αH^ and dimer2^βH^, Table 1 shows the experimental ${}^{3}$J_HH_ coupling constants and the computed values from the LEUS simulations for each residue. For the trimer and dimer^αC^, the corresponding values are given in Table 2.

**Figure 3:**
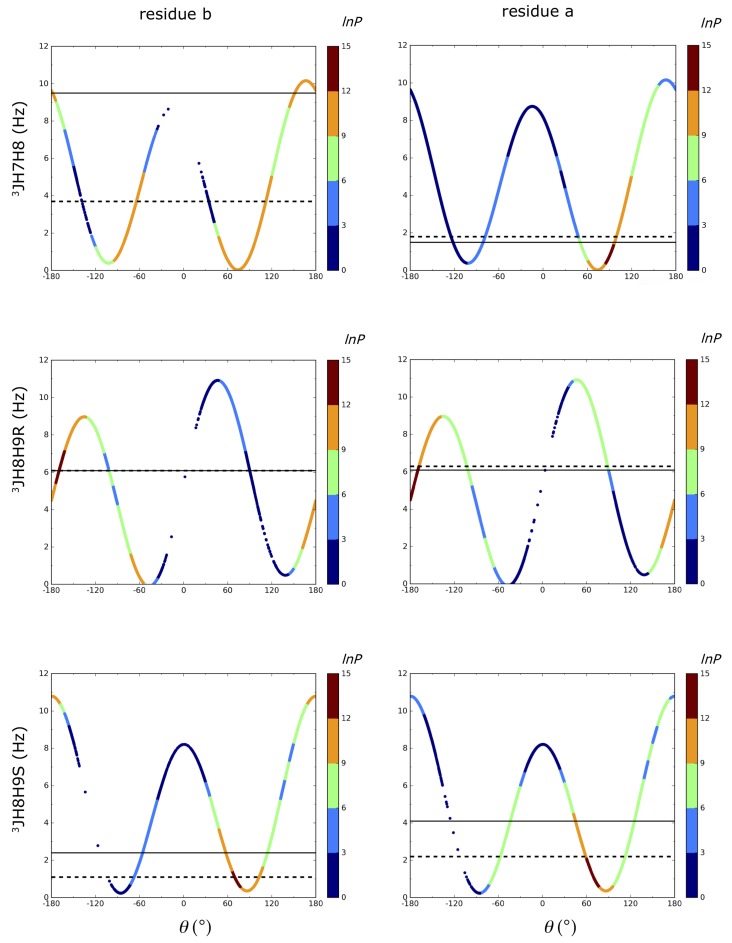
θ vs. calculated ${}^{3}$J_H7H8_,${}^{3}$J_H8H9R_, and ${}^{3}$J_H8H9S_ couplings from LEUS simulations for dimer1^αH^. The first column represents the first non-reducing residue b where the ω7 and ω8 are free and the second column is for residue a where they are part of the glycosidic linkage. Experimental and calculated ${}^{3}$J values are represented with solid and dashed horizontal lines, respectively. The colors on this Karplus curve indicate the preferred sampling after unbiasing of the LEUS simulations. In the unbiasing procedure, LEUS occurrences (P) are binned with 6${}^{\circ }$ grid spacing. Negative values of lnP are set to zero. The definition of the θ for each J value is given in Figure 1 and Equation (4).

**Figure 4:**
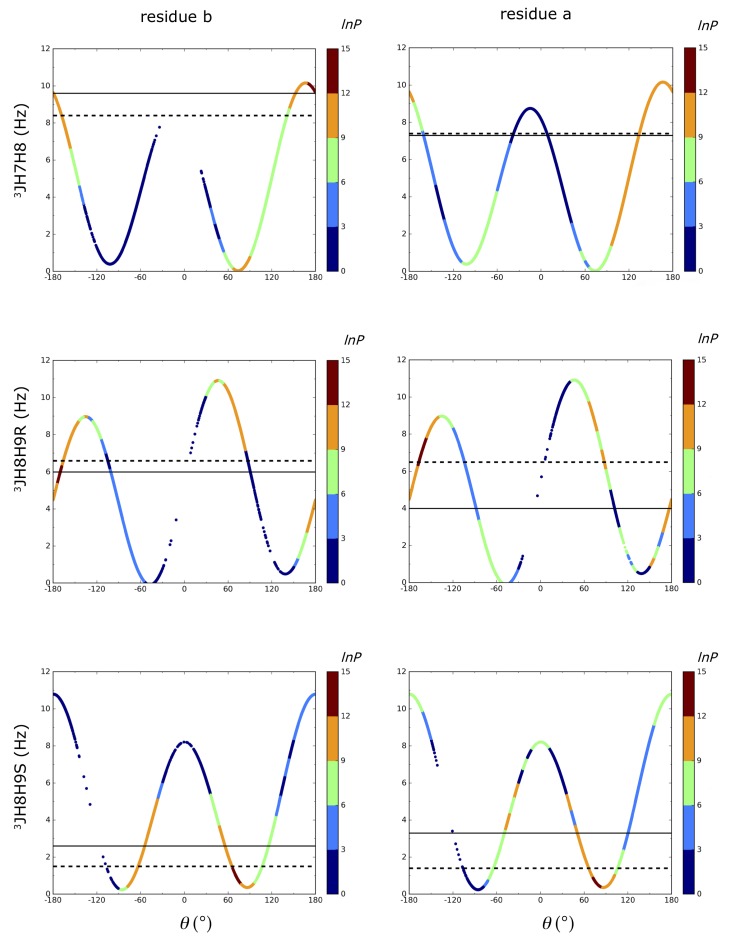
θ vs. calculated ${}^{3}$J_H7H8_,${}^{3}$J_H8H9R_, and ${}^{3}$J_H8H9S_ couplings from LEUS simulations for dimer2^βH^. The first column represents the non-reducing residue b, where the ω7 and ω8 are free and the second column is residue a where they are part of the glycosidic linkage. Experimental and calculated ${}^{3}$J values are represented with solid and dashed horizontal lines, respectively. The colors on this Karplus curve indicate the preferred sampling after unbiasing of the LEUS simulations. In the unbiasing procedure, LEUS occurrences (P) are binned with 6${}^{\circ }$ grid spacing. Negative values of lnP set to zero. The definition of the θ for each J value is given in Figure 1.

${}^{3}$J_H6H7_ coupling constants for all systems was calculated to be lower than 1.5 Hz which were also reported in NMR. ${}^{3}$J_H7H8_ couplings showed the maximum deviation for residue b in dimer1^βH^ system with 5.8 Hz deviation. This can be inspected from Figure 3. Although the energetically most favored region was calculated to be around 180${}^{\circ }$, the conformation around 60${}^{\circ }$ was also highly populated and this resulted in a significant decrease in the average ${}^{3}$J_H7H8_ coupling down to 3.7 Hz. In our LEUS simulations, the nonglycosylated ${}^{3}$J_H7H8_ coupling of residue b in the dimers and residue c in the trimer is calculated as 3.7 and 2.5 Hz for α ending dimers (dimer1^αH^, dimer3^αC^) and 4.7 Hz for trimer1^αC^. However, in the β ending dimer2^βH^, it was calculated as 8.4 Hz. As can be seen from the shape of the Karplus relations of the ${}^{3}$J_H7H8_ coupling in Figure 3 and Figure 4, the two extrema at 60${}^{\circ }$ and 180${}^{\circ }$ contribute values of ${}^{3}$J_H7H8_ coupling lower than 2 Hz and higher than 9 Hz, respectively for the α ending systems, resulting in an average value of the two. This is in contrast to the high value of nonglycosylated ${}^{3}$J_H7H8_ coupling in dimer2^βH^ which had a lower population around 60${}^{\circ }$, leading to a dominance of the 180${}^{\circ }$ region, giving a high J-value. However, reported values from NMR suggest that ${}^{3}$J_H7H8_ coupling on the nonglycosylated chain is ∼ 9.5 Hz in all α and β ending systems. Possibly, the nonglycosylated chains are slightly too flexible in our simulations of the α ending systems.

If we turn our attention to the ${}^{3}$J_H7H8_ coupling constant on the glycosylated chain (residue a), all systems are in agreement with NMR values with a maximum of 0.3 Hz deviation. While α dimer1^αH^ and α dimer3^αC^ give ${}^{3}$J_H7H8_ values of 1.8 and 1.0 Hz, β dimer2^βH^ gives 7.4 Hz, in close agreement with the experimental values. This ${}^{3}$J coupling constant shows the main difference between having an α and β terminus, emphasizing the effect of the stereochemistry of the reducing end on the glycosidic dihedral angle preference. In Figure 2 and the colors of Figure 3 and Figure 4, this is reflected by the increased preference for conformations with ω8≈ 180${}^{\circ }$.

Systems were largely in agreement with the experimental ${}^{3}$J${}_{H8H9R/S}$ values with a maximum deviation of 2.5 Hz for residue a in dimer2^βH^. The average absolute deviation over eight ${}^{3}$J${}_{H8H9R/S}$ coupling constants in dimer1^αH^ and dimer2^βH^ amounts to 1.1 Hz. No significant difference between α and β-terminated systems in ${}^{3}$J${}_{H8H9R/S}$ values were found in the LEUS simulations which were also reported in NMR experiments.

To complement the J-coupling data of the tetramer, coupling constants of tetrasialic acid were derived from a published 850 MHz proton spectrum as well as from J-resolved 600 MHz experiments (see Figures S1 and S2 in the supplementary material). A high-order spin system of H9c H8c overlapping with H9b was cross-checked by spin simulation [20].

The full ${}^{2}$J and ${}^{3}$J coupling assignments from NMR experiments of the tetramer are reported in Table S1 in the supplementary material. The comparison of the ${}^{3}$J${}_{H6H7}$, ${}^{3}$J${}_{H7H8}$, and ${}^{3}$J${}_{H8H9R/S}$ coupling constants with LEUS simulations of the tetramer are represented in Table 3 and in Figures S3 and S4. ${}^{3}$J${}_{H6H7}$ couplings show maximum deviation in residue d with 1.1 Hz. ${}^{3}$J${}_{H7H8}$ coupling at the free, non-glycosylated end in residue d is calculated as 9.8 which is in agreement with the NMR findings. Only residue a with a β-terminus showed a 3.6 Hz deviation. If it is assumed to be similar to dimer2^βH^, ${}^{3}$J${}_{H7H8}$ would have to be bigger than 7 Hz. The reason for not capturing the higher value might be due to strong interactions with the other residues, resulting in a different conformational preference of the tetramer or it can be due to a too pronounced sampling of the lower extreme of the Karplus curve for ω8 around 60${}^{\circ }$. For the ${}^{3}$J${}_{H8H9R/S}$ coupling constants in the tetramer, the highest deviation is seen at the second residue (c) with 6.5 Hz deviation. NMR showed values of 5.9 and 4.1 for ${}^{3}$J${}_{H8H9R}$ and ${}^{3}$J${}_{H8H9S}$ while LEUS calculations gave a value at the two extrema of the Karplus relation. This might be an indication of poor sampling of one of the two conformations.

### 2.3. Hydrogen Bonding

The occurrence of H-bonds was reweighed to the unbiased ensemble for the LEUS simulations. H-bonds with occurrences larger than 2% are reported in Table 4 for dimer and trimer systems.

Hydrogen bonds between the oxygen of the carboxyl group and the hydroxyl group of either the free or the glycosylated side chain were found to be highly populated in these systems. Dimer2^βH^ showed a more prominent intermolecular hydrogen-bonding pattern, as was reported by Azurmendi et al. as well [21].

### 2.4. MD Simulations of Tetramer with NOE Restraining

The CBCANH and SOLEXSY experimental studies of Battistel et al. indicated that the tetramer shows an H-bond for each of the first three residues of the molecule between HN5-O8 at 263 K [11]. They suggested that conformations with this transient H-bond pattern can be in equilibrium with other conformations at higher temperatures. In our LEUS simulations of the tetramer, this inter-residue hydrogen bond was not prominent. To obtain the related conformation, we have performed two additional simulations in which we imposed instantaneous distance restraints derived from the NOE data at 263 K and 300 K. In addition to NOE’s, Battistel et al. reported ${}^{4}$J_H7-C2_ coupling constants (also called W-couplings) via HSQMBC with 0.8 and 1.5 Hz for the second and the third residue, respectively. This spin coupling is measurable when C2 and H7 are in a W-shaped arrangement (C2-O6-C6-C7-H7 is quasi-coplanar) such that a C6-C7 conformation orienting H6 approximately anti to O7 is highly populated in solution. This long-range coupling was also reported as 0.8 Hz in the monomeric form of β-sialic acid by Klepach et al. [22]. We have used two additional dihedral restraining potentials for these two residues to sustain ${}^{4}$J_H7-C2_ couplings by restraining the H7-C7-C6-H6 dihedral angle to approximately 90${}^{\circ }$.

Battistel et al. have generated structures based on three H-bond restraints and setting two H7-C7-C6-H6 dihedral angles at residues c and b at approximately 90${}^{\circ }$. By using only these observations they created models resembling a 2${}_{4}$ and 1${}_{4}$ helix. Their NOE’s were complementing the static 2${}_{4}$ helix model with a higher percentage (71%) in which the exo-anomericity was not satisfied. Therefore, we aimed to build conformation(s) of oligosialic acids from the experimental data (instead of imposing experimental data on static models). For this aim, we have combined all experimental findings for a model with 2 dihedral angle restraints for the ${}^{4}J$-couplings, 3 distance restraints for hydrogen bonding and 82 NOE-derived distance restraints. Simulations were performed at 263 K and 300 K.

The energetic barriers between possible ring conformers of sialic acid are relatively shallow. The free energy penalty of the ring conformational change from the most stable conformer ${}^{2}$C${}_{5}$ to the closest alternative conformations ${}^{4,O}$B/${}^{O}$S${}_{3}$ and ${}^{4}$S${}_{2}$ was calculated to be less than 10 k${}_{B}$T [23]. As imposing restraints might disrupt the chair conformation, we have checked the ring puckering parameters and all sialic acid residues stayed at their energetically favored ${}^{2}$C${}_{5}$ chair conformation. Ring puckering parameters were calculated and are reported as ϕ vs. θ for all residues in Figure S5. Idealized ring puckering coordinate parameters are given in Table S4.

Our dynamical model resulted in compact conformations satisfying their NMR findings. These conformations are consistent with the exo-anomeric effect. The obtained set of conformations satisfied all NOE’s and H-bonds to within 0.1 Å (see Figure S6) and the two dihedral angles stayed at 70${}^{\circ }$ with a total of 880 kJ/mol restraining energy in both the 263 K and 300 K simulations. We have clustered the resulting ensemble by using an RMSD matrix with a 0.15 nm cutoff which resulted in six clusters [24]. 97% of the ensemble was clustered into a single cluster and the hydrogen bonding analysis of this cluster reveals a similar inter-residue pattern as in our LEUS simulation. Inter-residue hydrogen bonds between successive residues were observed between HN5-O1A/B, HO7-O1A/B, and HO9-O6 for 95%, 90%, and 37% of simulation time, respectively. Among the rest of the clusters, only one of them, with 15 structures out of 8000, showed the successive HN5-O8 H-bonding pattern as suggested by Battistel et al. (see Figure S7). No significant differences were observed between 263 K and 300 K. Note that the experiments were performed at 263 K, but also at this lower temperature, the HN5-O8 H-bond was only rarely observed in our simulations, in spite of the restraints.

### 2.5. Helical Conformation?

Thermodynamic integration (TI) was used to compute the free-energy difference between conformation satisfying NOE’s and ${}^{4}J$-couplings (state A) and conformations with three consecutive H-bonds that are observed in the NMR experiment [11] (state B). The initial structure for state A was taken from the end of the simulation with NOE restraining. Then, with the coupling parameter λ, three hydrogen bond restraints were added. By integrating over the derivative of the Hamiltonian with respect to λ, we obtain the free-energy difference (ΔG${}_{A\to B}$) as 81.5 kJ/mol. Then, with one step perturbation, we calculated the free energy of releasing the H-bond restraints for the conformations of state B by using Equation (8) as -15.2 kJ/mol (see Materials and Methods and Figure S8). Based on the free-energy calculations, the proposed helical structure with three consecutive hydrogen bonds between O8 and HN5 is unfavorable by about 65 kJ/mol. This 25 k${}_{B}$T shows that the conformation with three consecutive hydrogen bonds is not a thermodynamically accessible state for the tetramer.

**Figure 5:**
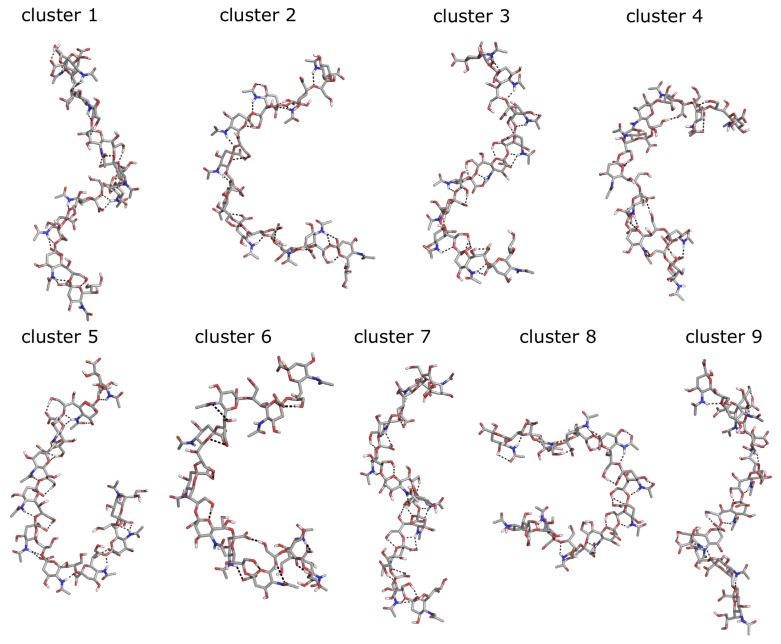
Central members of the most populated clusters of the jointly clustered configurations of α-2,8-decasialic acid from plain MD and H-bond restrained simulations. Clusters 1–3 and 5–9 contained conformations that originated from the restrained simulations. Cluster 4 originated from the plain MD simulations.

To check if the energetic gain of the Hydrogen bonds may counteract the entropic loss of conformational freedom for larger oligomers, we have constructed a decameric structure as well. By using our free-energy landscapes, we have constructed an decamer of α-2,8-linked-polysialic acid by setting the glycosidic dihedral angles to their respective lowest free-energy states (A, B and C) in Figure 2. For each lowest energetic state, a set of structures was generated (81 decameric structures) by setting ϕ and ψ dihedral angles for the four linkages in the pentamer system and repeating this to a decamer. Among those 81 structures, the two non-clashing lowest energetic ones were selected and equilibrated to be used as the initial structure for 100 ns simulations with plain MD and with hydrogen bond restraints on the O8-HN5 distance. In the plain MD simulations, the O8-HN5 distances fluctuate in the range of 3.7–5.0 nm and conformations largely resemble random coils. In contrast, the restraint simulations showed different helical patterns. We have jointly clustered the configurations from plain MD and H-bond restrained simulations with a 0.25 nm cutoff. No overlap of the most observed conformations was observed. Of the first nine clusters, clusters 1–3 and 5–9 contained conformations that originated from the restrained simulations. Cluster 4 originated from the plain MD simulations, with all remaining conformations forming smaller undefined clusters. Figure 5 shows the central member structures of the first nine clusters. Helical patterns are observed in clusters 1, 3, 7 and 9 amounting to 37% of the restrained simulations. The plain MD simulations, however, confirm that helical structures with O8-HN5 hydrogen bonds are also not thermodynamically likely in decasialic acid. Along with the relaxation NMR study of Henderson et al., our results support a flexible conformation for polysialic acid. Another supporting NMR study is from Hanashima et al. [25] who found significant differences in the interresidue transferred NOE correlations of bound and free conformations of trisialic and hexasialic acid units suggesting distinct conformations. This shows that highly unfavorable conformations may still be stabilized when polysialic acid is bound to an antibody or within a glycoprotein environment.

Finally, we have modeled an antibody-decasialic acid complex by using the crystal structure of the single-chain variable fragment of an anti-polysialic acid antibody, which was in a complex with octasialic acid (see Figure S9). We created a trajectory from all the structures that belong to cluster 4 (which is the most populated cluster originating from plain MD simulations) and aligned this to the octasialic acid in the crystal structure. It shows that the alternative ensemble of decasialic acid obtained in this study can fit well to the crystal structure of the anti-polysialic acid antibody.

## 3. Materials and Methods

### 3.1. NMR Experiments

Tetrasialic acid was obtained from Hycultec (Cat No C-7005-10) and measured in D${}_{2}$O at 300 K at a concentration of 3.5 mM. For NMR experiments the Bruker Topspin 3.5 PL 6 Software Suite was used on a Bruker Avance III console with a 600 MHz magnet. Assignments of proton and carbon chemical shifts and the 850 MHz ${}^{1}$H NMR spectrum were available from previous work [26].

For JRES experiments, a predefined Bruker parameter set (PROF_JRES) that employs the jresgpprqf pulse program with a a time domain for F2 of 32k and F1 200 data points (FIDRES F2 = 0.3 Hz and F1 = 0.8 Hz) was used. For HSQC, the pulseprogram hsqcedetgp and for COSY cosygpqf was employed.

### 3.2. MD Simulation Settings

In this study, we have used the six sialic acid systems represented in Figure 1. These involved three dimeric systems with different anomericity and end-groups at the reducing end, one trimer, one tetramer, and one decamer. All MD simulations were performed using the GROMOS11 biomolecular simulation package (http://www.gromos.net) [27] and the 53A6${}_{GLYC}$ carbohydrate parameter set [15]. We have previously introduced minor modifications to the original 53A6${}_{GLYC}$ carbohydrate parameter set for the sialic acid building block to ensure consistency with the rest of the force field [18]. Initial structures of the studied units were modeled in the molecular operating environment (MOE) [28] by setting their glycosidic dihedral angles to their respective free-energy minima which have been previously reported [18]. Short energy minimization was performed using the steepest-descent algorithm in a vacuum. The compounds were placed in a periodic cubic water box with simple point charge (SPC) water [29] molecules and initialized with a 1.4 nm minimum distance of the solute to the box walls. With position restraints on the solute atoms, the system was further relaxed by a steepest descent minimization. Then, the systems were equilibrated with initial random velocities generated from a Maxwell–Boltzmann distribution at 60 K and heated up to 300 K in five discrete steps. While heating up the system, position restraints on the solute atoms were reduced from 2.5 $\times 10^{4}$ to 0.0 kJ mol${}^{-1}$nm${}^{-2}$.

The production simulations were performed at a constant temperature of 300 K and a constant pressure of 1 atm using a weak coupling scheme [30] for both temperature and pressure with coupling times $\tau_{T}$ = 0.1 ps and $\tau_{P}$ = 0.5 ps, respectively with isothermal compressibility of 4.575 × $10^{-4}$ kJ${}^{-1}$ mol nm${}^{3}$. Newton’s equations of motion were integrated using the leapfrog scheme [31] with a time step of 2 fs. The SHAKE algorithm [32] was used to maintain the bond lengths at their optimal values. Long-range electrostatic interactions beyond a cutoff of 1.4 nm were truncated and approximated by a generalized reaction field [33] with a relative dielectric permittivity of 61 [34]. Nonbonded interactions up to a distance of 0.8 nm, were computed at every time step using a pairlist [35] that was updated every 5 steps. Interactions up to 1.4 nm, were computed at pairlist updates and kept constant in between.

The GROMOS++ software [36] is used for time series analysis. A geometrical criterion was used to identify hydrogen bonds if a hydrogen-acceptor distance is smaller than 0.25 nm and the donor-hydrogen-acceptor angle is larger than 135${}^{\circ }$.

### 3.3. Creating Biased Potentials with Local Elevation and Sampling with Umbrella Sampling

**Figure 6:**
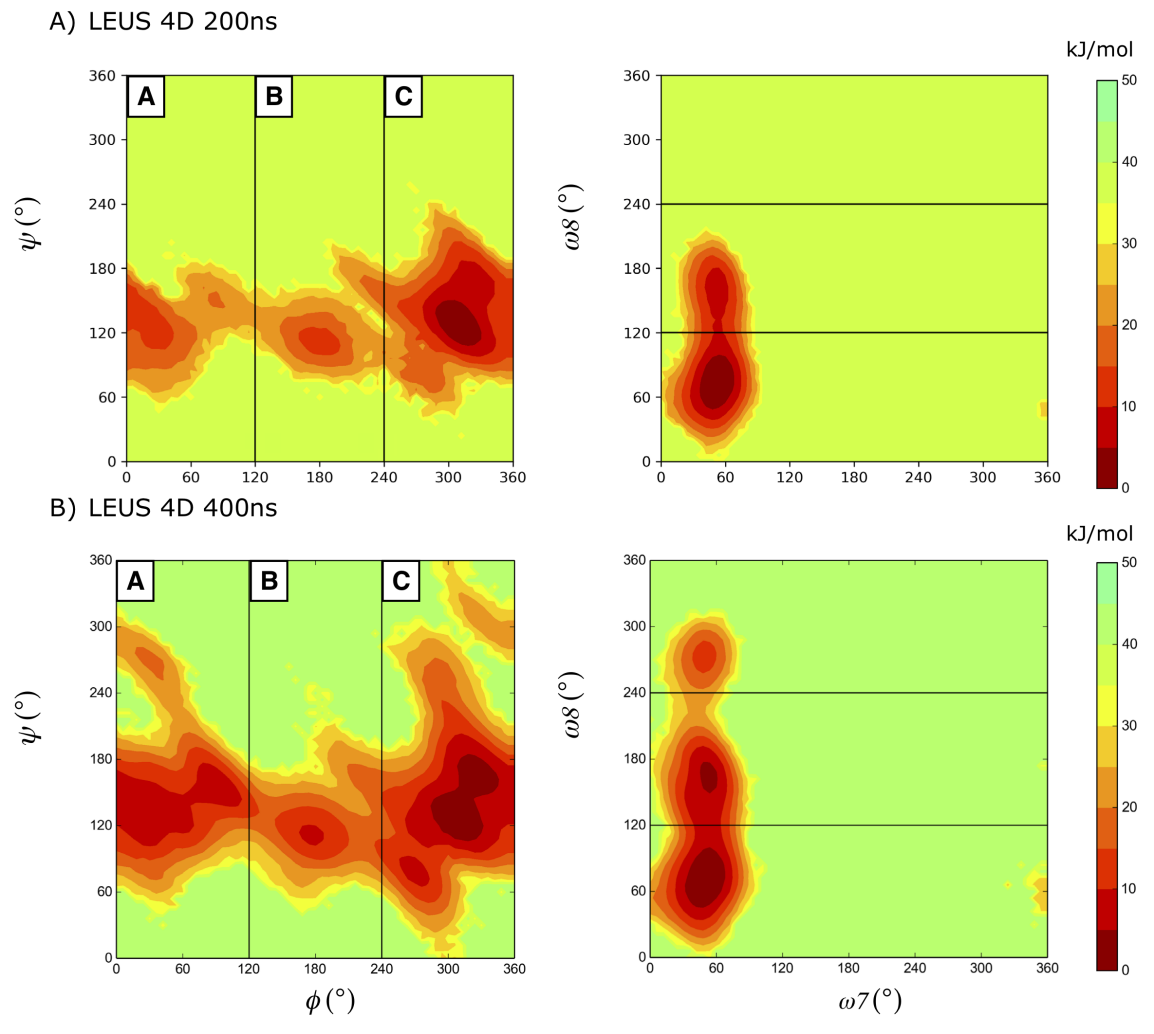
Conformational sampling for dimer3^αC^ in a 4D local elevation buildup simulation after 200 ns (**A**) and 400 ns (**B**).

For each system, unbiased MD simulations were carried out for 100 ns after equilibration. In addition to unbiased simulations, an enhanced sampling method, local elevation with umbrella sampling (LEUS) [37,38] was applied. For the local elevation potential build-up, the glycosidic dihedral angles of the systems were used. For a detailed description of the methodology, see our previous work in Ref. [18]. In short, in the LEUS method dihedral angles are binned in $N_{g}$ = 36 bins, and a biasing potential width of σ = 360${}^{\circ }$/$N_{g}$ was used with a force constant increment of *c* = 0.005 kJ mol${}^{-1}$. In the current work, the studied systems have four conformational degrees of freedom along their 2→8 glycosidic linkage. To ensure a near-to-complete sampling along the glycosidic linkage, two different two-dimensional potentials with ϕ, ψ and ω8, ω7 were used. For definitions of the dihedral angles see the caption of Figure 1. Local elevation potentials were only built up for dimer systems (dimers 1–3). Dimer2 and dimer3 potentials were used for umbrella sampling for the tetramer and dimer3 for the trimer. Two different end groups used in the dimer systems; OH group (dimer 1 and dimer 2) and OMe group (dimer 3), to compare the effect of the end group. In addition, different combinations of 3D and 4D potentials for the ϕ, ψ, ω8 and ω7 were checked but those attempts did not offer additional sampling. Although in principle a long four-dimensional build up along all four degrees of freedom (ϕ, ψ, ω8, and ω7) offers a complete sampling, it was not as efficient as using two different 2D potentials. Figure 6 shows the free energy landscape obtained after a four-dimensional build up of 200 and 400 ns for dimer3. Only after 400 ns the same coverage is achieved as with two 2D LE potentials (Figure 2C).

Therefore, after the optimization of the creation of the LE potentials two 2D LE potentials are built for the glycosidic linkage with $t_{LE}$ = 100 ns. These potentials are created for all the three dimer systems as they differ in type and anomericity of the end groups. In the US phase, the LE biased potentials were frozen and sampling was applied by using both 2D potentials. Then, umbrella sampling was applied to those potentials by saving trajectories every 0.1 ps for 100 ns to achieve statistical efficiency as discussed in Ref. [37].

The unbiased probability of any property *Q* can be obtained from the LEUS (biased) simulations through reweighing:

(1) $$P\left(Q^{\circ }\right)=\frac{\langle \delta \left(Q-Q^{\circ }\right)\exp\left[U_{LEUS}\left(Q\right)/k_{B}T\right]\rangle }{\langle \exp\left[U_{LEUS}\left(Q\right)/k_{B}T\right]\rangle },$$

where 〈〉 indicates an ensemble average of the biased LEUS simulation, $U_{\mathrm{LEUS}}$(Q) is the biasing energy at a particular value of *Q*, δ is the Kronecker delta function, *k_B_* is the Boltzmann constant and *T* is the absolute temperature. The corresponding free energies can be obtained from the calculated probabilities,

(2) $$G\left(Q\right)=-k_{B}T\ln P\left(Q\right).$$

### 3.4. Analysis

For dimer systems (1–3) two free-energy maps, G(ϕ,ψ) and G(ω8,ω7) were created from the LEUS simulations after reweighing of the biased energy with Equations (1) and (2). The global minimum of each map represents the lowest free-energy conformation which is set to 0 kJ/mol and the colormap is drawn using a 5 kJ/mol contour. A more detailed explanation for the construction of the free-energy maps can be found in the methods section of Ref. [18].

### 3.5. ^3^J-coupling Constants and NOE Calculations

Simulations are compared with NOE data and ${}^{3}$J-coupling constants. Aliphatic carbon atoms are treated as united atoms in the GROMOS force field. Therefore, virtual atomic positions for prochiral CH_2_ (C3 and C9), for CH (C4, C5, C6, C7, and C8) and pseudo atomic positions for CH_3_ (C5A) were used to calculate interproton distances. Upper bound corrections are applied by centre averaging approach [39] and reported in Tables S2 and S3 (using for prochiral CH_2_ 0.09 nm and for CH_3_ 0.1 nm; no corrections are added for CH and explicit HN hydrogen atoms). For the NOE analysis, averaging is performed as $<r^{-6}{>}^{-1/6}$ [40].

${}^{3}$J-coupling constants can be related to a torsional angle through the Karplus relation. Coupling constants related to glycosidic dihedral angles are ${}^{3}$J${}_{H8H9R/S}$, ${}^{3}$J${}_{H7H8}$, and ${}^{3}$J${}_{H6H7}$ corresponding to ω9, ω8, and ω7 dihedral angles, respectively (Figure 1). ${}^{3}$J${}_{H6H7}$ and ${}^{3}$J${}_{H7H8}$ were calculated from MD and LEUS simulations using the Haasnoot Equation (3) which involves a modification to the Karplus relation, to include the effect of electronegativity of neighboring groups [41].

(3) $$\begin{array}{c} {}^{3}J_{HCCH}=P_{1}cos^{2}\theta +P_{2}cos\theta +P_{3}+\sum \Delta \chi_{i}[P_{4}+P_{5}cos^{2}(s_{i}\theta +P_{6}|\Delta \chi_{i}|)]. \end{array}$$

Here, the sum runs over the four substituents, $\Delta \chi_{i}=\Delta \chi^{\alpha }+P_{7}\sum \Delta \chi^{\beta }$ and s${}_{i}$ is the direction coefficient which is +1 for “positive” and −1 for “negative” substituents defined by their orientation relative to the attached α or β atom. Parameters P1–P7 used in Haasnoot equation are: 13.7, −0.73, 0, 0.56, −2.47, 16.9, and 0.14, respectively.

For prochiral protons, ${}^{3}$J${}_{H8H9R}$ and ${}^{3}$J${}_{H8H9S}$ values were calculated from the following equation which was derived by DFT methods [42]:

(4) $$\begin{array}{cc} {}^{3}J_{H8H9R} & =5.08+0.47cos\omega +0.9sin\omega -0.12cos2\omega +4.86sin2\omega ,\;\omega =\omega 9\\ {}^{3}J_{H8H9S} & =4.92-1.29cos\omega +0.05sin\omega +4.58cos2\omega +0.07sin2\omega ,\;\omega =\omega 9-120^{\circ }. \end{array}$$

### 3.6. Simulations with Distance Restraints

To study the possible conformations under the condition that available experimental data is fulfilled, we performed additional simulations with full harmonic distance restraints. In the restraint tetramer simulations, 82 instantaneous NOE restraints were applied [43] with a force constant of 500 kJ mol${}^{-1}$nm${}^{-2}$ and 3 instantaneous restraints on HN5-O8 pairs with a force constant of 2500 kJ mol${}^{-1}$nm${}^{-2}$ to satisfy hydrogen bonding. The NOE distance restraints used are listed in Tables S2 and S3 in the Supplementary Materials. Additionally, 2 instantaneous dihedral angle restraints were included with a force constant of 100 kJ mol${}^{-1}$degrees${}^{-2}$ for residues c and b at H7-C7-C6-H6 at 90${}^{\circ }$ to fulfill ${}^{4}$J${}_{H7-C2}$ coupling. Since the hydrogen bonding evidence and the NOE data experiments [11] were conducted at 263 K we simulated the tetramer at 263 K. An additional simulation with the same parameters at a temperature of 300 K was run to check the system at a temperature closer to physiological conditions. The conformations that are observed in the restraint simulations were further used as an initial structure for the free-energy calculation. Thereby, to the best of our knowledge, this is the first time where a quantitative conformational analysis was made for proposed polysialic acid structures and models.

### 3.7. Thermodynamic Integration and One-Step Perturbation

The change from a conformation satisfying experimental NOEs and ${}^{4}$J-coupling constants (state A) to a state B in which additionally three H-bonds are enforced, is performed with thermodynamic integration (TI). The path from state A to B is defined by a scaling parameter λ, where the Hamiltonian (H) at λ=0 describes state A and at λ=1 describes state B. The free energy difference between state A and B can be extracted by numerical integration of ${\langle \partial H/\partial \lambda \rangle }_{\lambda }$ as a function of λ with the following equation [44]:

(5) $$\Delta G_{A\to B}=G_{B}-G_{A}=\int_{0}^{1}{\langle \frac{\partial H}{\partial \lambda }\rangle }_{\lambda }d\lambda ,$$

where the angular brackets denote the ensemble average of the derivative of H(λ) with respect to λ. In state A, the force constant on the three hydrogen-bond restraints is 0, but the derivative of H still depends on the unrestrained distance between the atoms. This leads to instabilities in λ-derivatives during the perturbation. This is avoided by using a soft bond potential energy term which is introduced with a softness term (S(r,λ)) [45]:

(6) $$U\left(r,\lambda \right)=\frac{1}{2}\left[\left(1-\lambda \right)\frac{k^{A}}{S^{A}\left(r,\lambda \right)}+\lambda \frac{k^{B}}{S^{B}\left(r,1-\lambda \right)}\right]{\left[r-\left[\left(1-\lambda \right)r^{0A}+\lambda r^{0B}\right]\right]}^{2},$$

where the softness term for state X is

(7) $$S{}^{X}\left(r,\lambda \right)=1+\alpha \lambda {\left(r-r^{0X}\right)}^{2},$$

with α a unitless softness parameter set to 250. The force constants $k^{A}$ and $k^{B}$ were set to be $k^{A}=0$ and $k^{B}$ = 5 × 10${}^{4}$ kJ mol${}^{-1}$nm${}^{-2}$ and the optimal bond length was set to $r^{0A}=r^{0B}=0.25\;\mathrm{nm}$. First equidistant λ values were used for the change between states A and B. Then, to achieve a smooth transformation, additional λ points were introduced. In total 12 λ points were first equilibrated for 50 ps, followed by a production run of 2 ns for each λ point sequentially. Initial configuration at a certain λ point were taken from the final configuration of the previous λ value. The coordinates and energies were stored every 0.2 ps.

Then, one-step perturbation was applied on the restrained ensemble to remove the hydrogen bond restraints (state C). State C represents an unrestrained ensemble, which only covers the phase space in which the hydrogen bonds are observed. The free energy difference between state B and state C is calculated using the Zwanzig formula [46]:

(8) $$\Delta G_{B\to C}=G_{C}-G_{B}=-k_{B}T\ln{\langle e^{-\left(H_{C}-H_{B}\right)/k_{B}T}\rangle }_{B},$$

where $k_{B}$ is the Boltzmann constant, *T* is the temperature, and the difference between the Hamiltonian of two states is the restraining energy ($H_{B}-H_{C}=U_{res}$ ). ${\langle \rangle }_{B}$ represents the ensemble average over all configurations generated during the simulation at state B which was run for 100 ns.

## 4. Conclusions

We have studied the conformational freedom of the α-2,8-linkage between two subsequent sialic acid monomers with an enhanced conformational searching and sampling method, LEUS. Our simulations showed that the NMR observations can be satisfied with a set of conformations that are very similar to the ones observed in our LEUS simulations. The largest deviations in terms of ${}^{3}J$-coupling constants are observed for the free non-glycosylated tail of the terminal residue in the C8-C9 sidearm, while the parameters determining the glycosidic linkage between two sialic acid units are excellently reproduced. Due to the extra degrees of freedom in the α-2,8-linkage, multiple conformations are accessible, as observed in the free-energy landscape of the dimers. However, the hydrogen bond that was previously suggested to support the helical conformation was found to be very transient in our restrained tetra-sialic acid simulations. The thermodynamic penalty of enforcing all three of these hydrogen bonds simultaneously was computed to be highly unfavorable, with about 25 k${}_{B}T$. While enforcing the hydrogen bonds in decasialic acid does lead to helical structures (up to 37%), such conformations are not observed in plain MD simulations of the same molecule. From the current work, we conclude that helical conformations, while possible, are unlikely to play a dominant role in free polysialic acid, but would need to be induced upon binding to a partner, requiring a considerable enthalpy-entropy compensation.

## Figures and Tables

**Table 1:** Experimental and calculated ${}^{3}$J_HH_ coupling constants for α-Neu5Ac-(2-8)-α-Neu5Ac-OH (dimer1^αH^) and α-Neu5Ac-(2-8)-β-Neu5Ac-OH (dimer2^βH^). Calculated values from LEUS simulations after unbiasing.

	dimer1^αH^			dimer2^βH^		
	Residue	NMR ^*a*^	LEUS	Residue	NMR ^*b*^	LEUS
${}^{3}$J_H6H7_ [Hz]	b	1.4 ± 0.1	1.4 ± 0.2	b	< 1.5	0.9 ± 0.1
	a	<1.0	0.9 ± 0.3	a	< 1.5	0.8 ± 0.1
${}^{3}$J_H7H8_ [Hz]	b	9.5 ± 0.9	3.7 ± 0.6	b	9.6 ± 0.5	8.4 ± 0.1
	a	1.5 ± 0.2	1.8 ± 0.1	a	7.3 ± 0.5	7.4 ± 0.1
${}^{3}$J_H8H9R_ [Hz]	b	6.1 ± 0.6	6.1 ± 0.1	b	6.0 ± 0.5	6.6 ± 0.1
	a	6.1 ± 0.6	6.3 ± 0.1	a	4.0 ± 0.5	6.5 ± 0.1
${}^{3}$J_H8H9S_ [Hz]	b	2.4 ± 0.2	1.1 ± 0.1	b	2.6 ± 0.5	1.5 ± 0.1
	a	4.1 ± 0.4	2.2 ± 0.5	a	3.3 ± 0.5	1.4 ± 0.1

^*a*^ Experimental values from Ref. [10]; ^*b*^ from Ref. [19].

**Table 2:** Experimental and calculated ${}^{3}$J_HH_ coupling constants for α-Neu5Ac-(2-8)-α-Neu5Ac-α-(2-8)-α- Neu5Ac-OMe (trimer1) and α-Neu5Ac-(2-8)-α-Neu5Ac-OMe (dimer 3^αC^). Calculated values from LEUS simulations after reweighing.

	trimer1^αC^			dimer3^αC^	
	Residue	NMR ^*a*^	LEUS	Residue	LEUS
${}^{3}$J_H6H7_ [Hz]	c	1.5 ± 0.2	1.0 ± 0.2	b	1.0 ± 0.1
	b	< 1.0	1.0 ± 0.3	a	0.9 ± 0.1
	a	< 1.0	1.0 ± 0.2		
${}^{3}$J_H7H8_ [Hz]	c	9.6 ± 1.0	4.7 ± 0.5	b	2.5 ± 0.5
	b	< 4.0	3.3 ± 0.7	a	1.0 ± 0.5
	a	< 4.0	6.4 ± 0.7		
${}^{3}$J_H8H9R_ [Hz]	c	n.d.	7.1 ± 0.1	b	5.9 ± 0.5
	b	n.d.	6.0 ± 0.1	a	5.9 ± 0.5
	a	n.d.	6.6 ± 0.1		
${}^{3}$J_H8H9S_ [Hz]	c	n.d.	2.8 ± 0.2	b	1.8 ± 0.5
	b	n.d.	1.2 ± 0.1	a	1.4 ± 0.5
	a	n.d.	0.9 ± 0.1		

^*a*^ Experimental values from Ref. [10].

**Table 3:** Experimental and calculated ${}^{3}$J_HH_ coupling constants for α-Neu5Ac-(2-8)-α-Neu5Ac-(2-8)-α- Neu5Ac-(2-8)-β-Neu5Ac-OH (tetramer^βH^). Calculated values from LEUS simulations after reweighing.

${}^{3}$J	tetramer^βH^		
	Residue	NMR ^*a*^	LEUS
${}^{3}$J_H6H7_	d	2.0 ± 0.1	0.9 ± 0.1
	c	< 1.0	0.8 ± 0.1
	b	< 1.0	0.6 ± 0.1
	a	< 1.0	0.6 ± 0.1
${}^{3}$J_H7H8_	d	8.9 ± 0.1	9.8 ± 0.0
	c	4.5 ± 0.1	1.7 ± 0.1
	b	2.7 ± 0.1	1.5 ± 0.4
	a	6.3 ± 0.1	2.7 ± 0.6
${}^{3}$J_H8H9R_	d	6.1 ± 0.1	6.9 ± 0.1
	c	5.9 ± 0.1	0.3 ± 0.6
	b	6.1 ± 0.1	5.1 ± 0.1
	a	4.3 ± 0.1	6.1 ± 0.1
${}^{3}$J_H8H9S_	d	2.5 ± 0.1	0.7 ± 0.3
	c	4.1 ± 0.1	10.6 ± 0.0
	b	5.4 ± 0.1	1.6 ± 0.1
	a	2.8 ± 0.1	1.2 ± 0.4

^*a*^ NMR experiments were conducted at 600 MHz and 800 MHz.

**Table 4:** Intra- and Inter-residue hydrogen bond occurrences along with the water bridges calculated from LEUS simulations for dimers and trimer. Reweighted populations higher than 2% are reported. ’-’ indicates that the hydrogen bond is not observed. ’n.a.’ indicates that the hydrogen bond not applicable for this system.

H-bond		System			
**Type**		**dimer1^αH^**	**dimer2^βH^**	**dimer3^αC^**	**trimer1^αC^**
Intra-residue	bHO8-bO1A/B	-	5.9%	5.3%	-
	aHO2-aO1A/B	-	25.2%	-	-
Inter-residue	aHO9-bO1A/B	38.4%	3.2%	19.4%	1.7%
	aHO7-bO1A/B	-	46.7 %	8.5%	14.9%
	aHO7-bO6	4.3%	-	-	14.7%
	aHO9-bO6	-	16.7%	8.2%	-
	aHN5-bO1A/B	-	-	3.1%	-
	aHO4-bO1A/B	-	7.7%	-	-
	bHO9-aO1A/B	-	40.0%	-	-
	bHO7-aO1A/B	-	2.4%	-	-
	cHO9-bO1A/B	n.a	n.a.	n.a.	3.8%
	cHO7-bO6	n.a.	n.a.	n.a.	6.3%
Water-bridge	aO6-bO8	-	11.5%	-	-
	bO6-bO2	-	56.5%	14.9%	-
	bHN5-bO2	-	17.9%	-	-

## Supplementary Materials

Supplementary materials are available online at https://www.mdpi.com/1422-0067/21/1/30/s1, Figures S1 and S2: Expansion plot of the 600 MHz J-resolved spectrum of tetrasialic acid, Figures S3 and S4: θ vs. calculated ${}^{3}$J_H7H8_,${}^{3}$J_H8H9R_ and ${}^{3}$J_H8H9S_ couplings from LEUS simulations for tetramer^βH^, Figure S5: Ring conformation of the residues in the tetramer, Figure S6: NOE violations from restrained simulation of tetramer, Figure S7: Representative structures from clusters to illustrate hydrogen bonding patterns from NOE-restrained simulation of the tetramer, Figure S8: TI and OSP plot, Figure S9: Representation of the anti-polysialic acid crystal structure of single-chain variable fragment in complex with octasialic acid, Table S1: 2D NMR assignments for tetramer^βH^, Tables S2 and S3: Intra and Inter-residue NOE restraining values, Table S4: Cremer-Pople parameters for the idealized ring conformations.

## Abbreviations

MD	Molecular Dynamics
NMR	Nuclear Magnetic Resonance
NOE	Nuclear Overhauser Effect
LEUS	Local Elevation Umbrella Sampling
