# Supplementary material for: Molecular Conformations of Di-, Tri-, and Tetra-α-(2→8)-Linked Sialic Acid from NMR Spectroscopy and MD Simulations
Source: Int J Mol Sci. 2019 Dec 19;21(1):30. doi: 10.3390/ijms21010030 (PMC6981865; doi:10.3390/ijms21010030)
Supplement: Supplementary file 1 [file ijms-21-00030-s001.pdf]

Supplementary Materials: Molecular conformations of di-, tri-, and tetra-α-(2$\rightarrow$8)-linked sialic acid from NMR spectroscopy and MD simulations

Aysegül Turupcu [1,†], Markus Blaukopf [2], Paul Kosma [2], and Chris Oostenbrink [1,*]

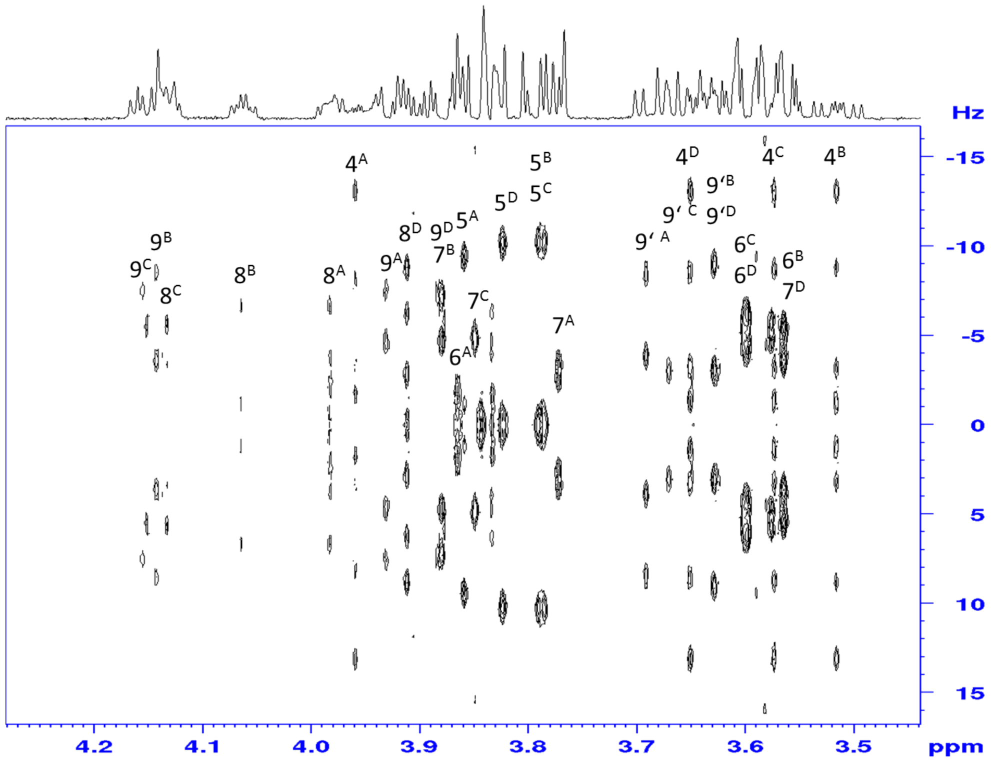

Figure S1. Expansion plot of the 600 MHz J-resolved spectrum of tetrasialic acid

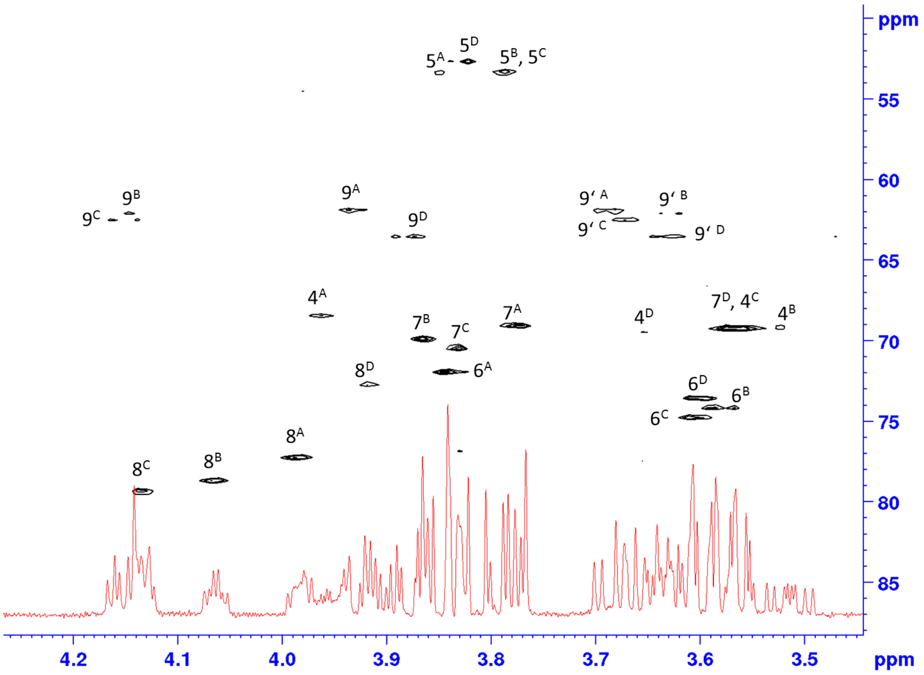

Figure S2. Expansion plot of the HSQC spectrum of tetrasialic acid recorded at 600 MHz

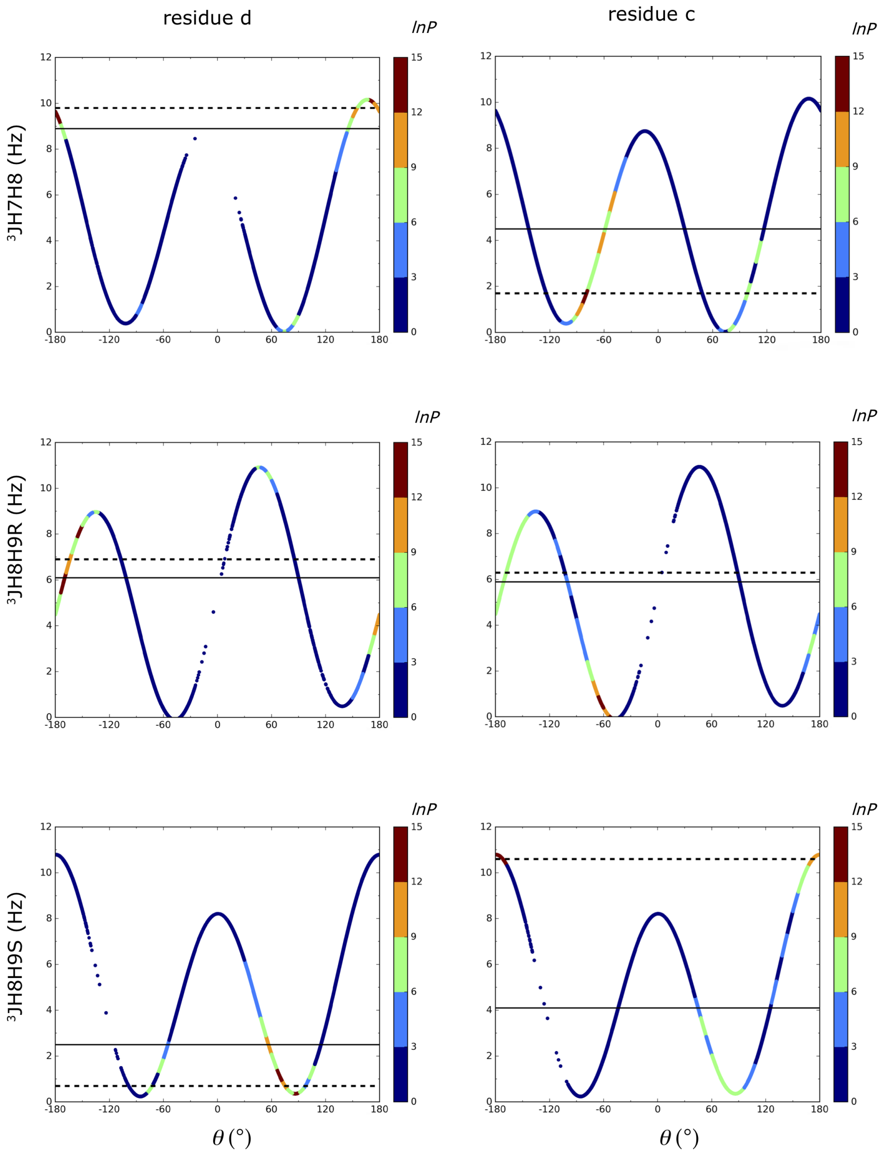

Figure S3. θ vs. calculated $^{3}J_{H7H8}$,$^{3}J_{H8H9R}$ and $^{3}J_{H8H9S}$ couplings from LEUS simulations for tetramer$^{\beta H}$. The first column represents the first non-reducing residue d where the $\omega 7$ and $\omega 8$ are free and the second column is for residue c where they are part of the glycosidic linkage. Experimental and calculated ^{3}J values are represented with solid and dashed horizontal lines, respectively. The colors on this Karplus curve indicate the preferred sampling after unbiasing of the LEUS simulations. In the unbiasing procedure, LEUS occurrences (P) are binned with 6° grid spacing. Negative values of lnP are set to zero.

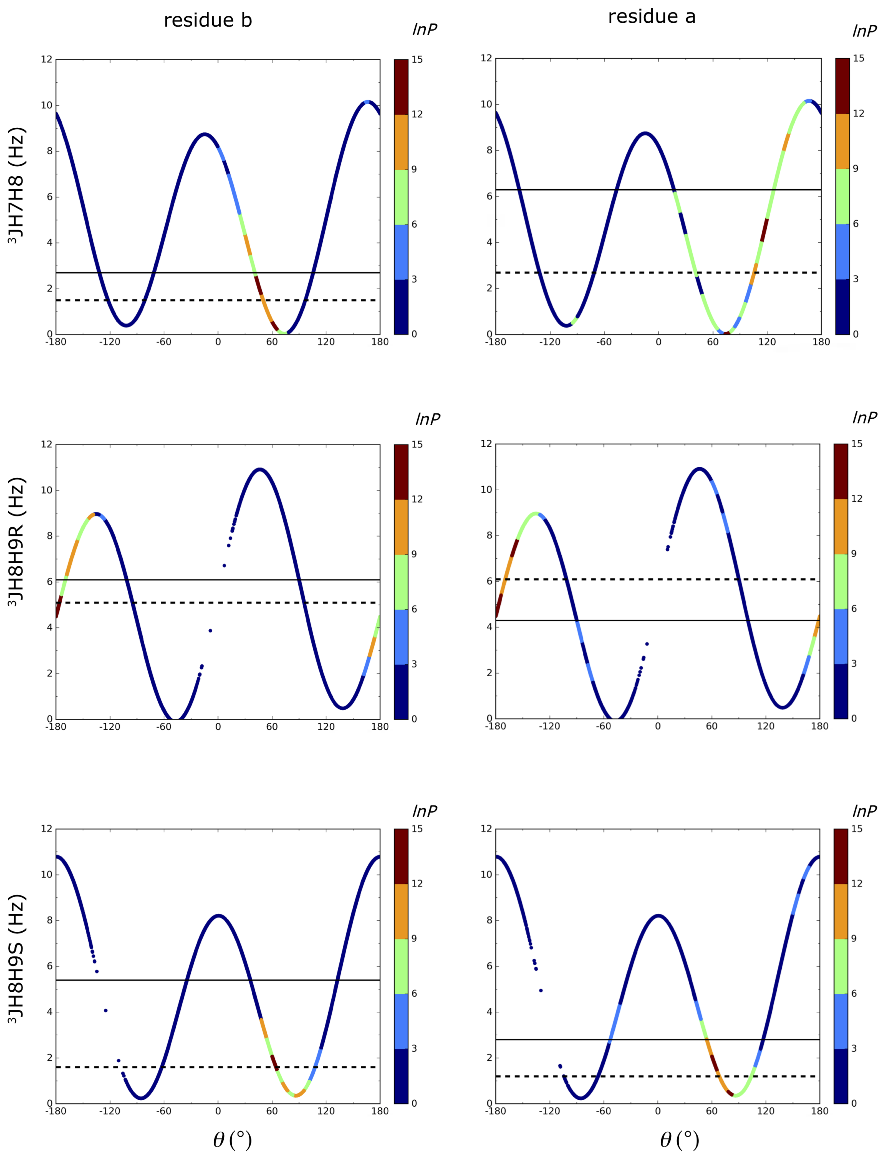

Figure S4. θ vs. calculated $^3J_{H7H8}$,$^3J_{H8H9R}$ and $^3J_{H8H9S}$ couplings from LEUS simulations for tetramer$^{\beta H}$. The first column represents residue b and the second column is for residue a where the $\omega 7$ and $\omega 8$ are part of the glycosidic linkage. Experimental and calculated ^{3}J values are represented with solid and dashed horizontal lines, respectively. The colors on this Karplus curve indicate the preferred sampling after unbiasing of the LEUS simulations. In the unbiasing procedure, LEUS occurrences (P) are binned with 6° grid spacing. Negative values of lnP are set to zero.

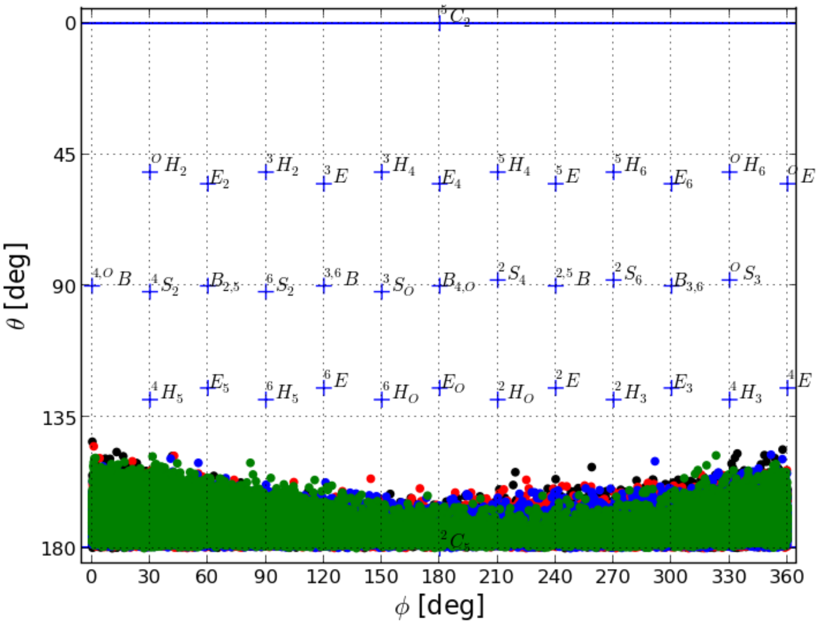

Figure S5. Ring conformation of the residues in the tetramer. All four residues, represented with different colors, stayed in $^{2}C_{5}$ chair conformation which is around θ = 180°.

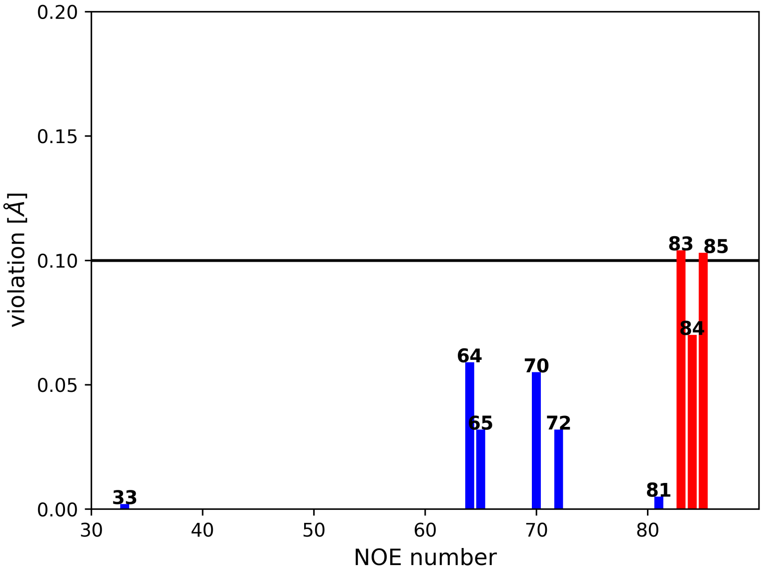

Figure S6. NOE violations from restrained simulation of tetramer. 82 NOE-derived upper bounds and 3 distances for hydrogen bonding (numbered with 83, 84 and 85 shown with red bars) were within 0.1 nm violation (black line). Numbers refers to NOE values used for different proton pairs. Full list of intra- and inter-residue NOE values are reported in Table S2 and S3 in the supplementary information, respectively. Annotated numbers are the ones showing small violation.

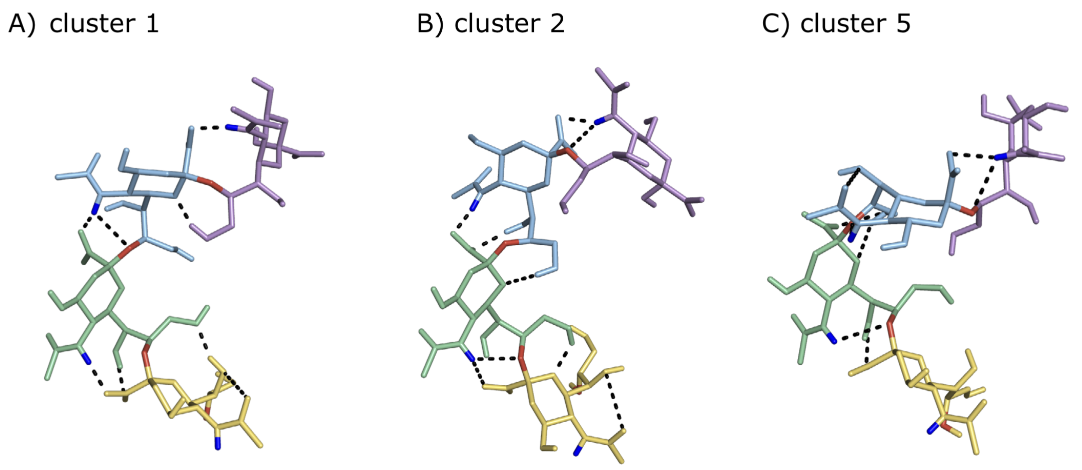

Figure S7. Representative structures from clusters to illustrate hydrogen bonding patterns from NOE-restrained simulation of the tetramer. A) Cluster 1 (the most populated cluster, 97 %) The H-bonds show same pattern with the studied dimers: HO9-O1A/B, HO8-O1A/B, HN5-O1A/B and HO9-O6; B) Cluster 2 (2nd most populated cluster); C) cluster 5 showing the repetitive HN5-O8 hydrogen bonding pattern (0.2 %)

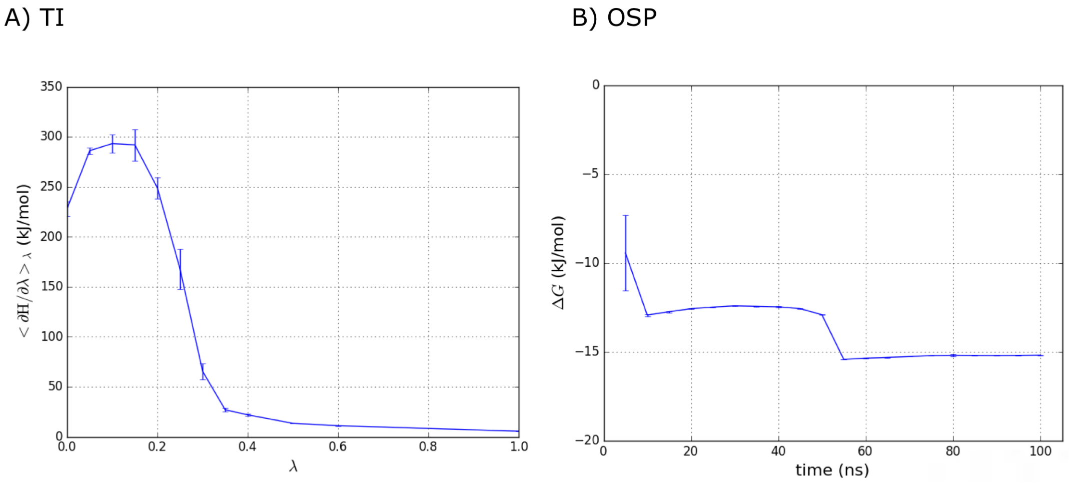

Figure S8. A) $\langle \partial \mathcal{H}/\partial \lambda \rangle_\lambda$ as a function of λ for thermodynamic integration between state A (free) and state B (hydrogen bonded) B) $\Delta G_{B \rightarrow C}$ as a function of time from one-step perturbation simulation

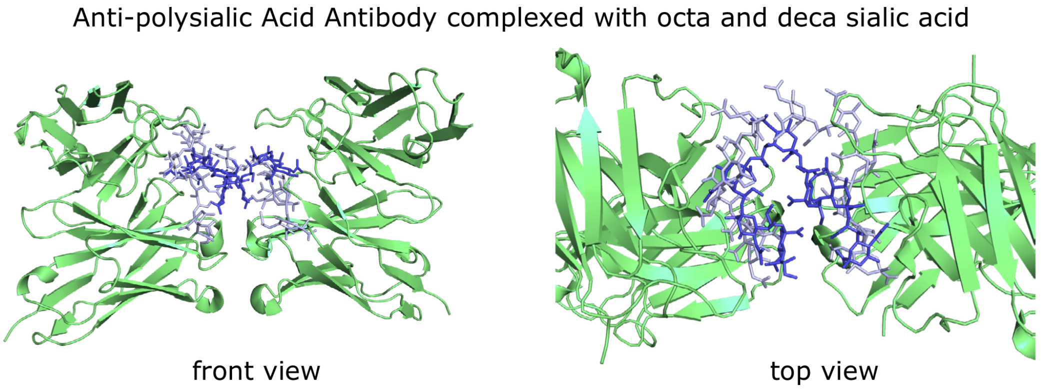

Figure S9. Representation of the anti-polysialic acid crystal structure of single chain variable fragment, scFv735, (green shown as cartoon) in complex with octasialic acid (dark blue in sticks) (pdb id: 3WBD[1]) and decasialic acid structure found in this study with high population (shown in light blue)

Table S1. 2D NMR assignments for tetramer$^{\beta H}$

Residue a	^{1}H	^{3}J	^{2}J	Residue b	^{1}H	^{3}J	^{2}J
3ax	1.76	$J_{3ax,4}$ 11.8	13.0	3ax	1.66	$J_{3ax,4}$ 12.2	12.2
3eq	2.21	$J_{3eq,4}$ 5.2	13.1	3eq	2.70	$J_{3eq,4}$ 4.4	12.4
4	3.99	$J_{3ax,4}$ 11.4, $J_{3eq,4}$ 5.2, $J_{4,5}$ 9.4		4	3.54	$J_{3eq,4}$ 4.5, $J_{3ax,4}$ 12.0, $J_{4,5}$ 10.1	
5	3.87	n.d.*		5	3.81	$J_{4,5}$ 10.3, $J_{5,6}$ 10.3	
6	3.86	n.d.*		6	3.59	$J_{5,6}$ 10.2, $J_{6,7} < 1.0$	
7	3.79	$J_{7,8}$ 6.3, $J_{7,6} < 1.0$		7	3.89	$J_{7,8}$ 2.7	
8	4.00	n.d.		8	4.09	$J_{8,7}$ 2.8, $J_{8,9b}$ 6.2	
9a	3.96	$J_{9a,8}$ 2.8	12.3	9a	4.17	$J_{8,9a}$ 5.4	
9b	3.73	$J_{9b,8}$ 4.3	12.3	9b	3.65	$J_{8,9b}$ 6.1	12.0
**Residue c**	**^{1}H**	**^{3}J**	**^{2}J**	**Residue d**	**^{1}H**	**^{3}J**	**^{2}J**
3ax	1.71	$J_{3ax,4}$ 12.2	12.3	3ax	1.76	$J_{3ax,4}$ 12.1	12.3
3eq	2.68	$J_{3eq,4}$ 4.5	12.3	3eq	2.77	$J_{3eq,4}$ 4.8	12.4
4	3.59	$J_{3eq,4}$ 4.2, $J_{4,5}$ 10.1		4	3.67	$J_{3eq,4}$ 4.5, $J_{3ax,4}$ 11.7, $J_{4,5}$ 10.0	
5	3.81	$J_{4,5}$ 10.2, $J_{5,6}$ 10.3		5	3.85	$J_{4,5}$ 10.2, $J_{5,6}$ 10.2	
6	3.62	$J_{6,5}$ 10.6, $J_{6,7} < 1.0$		6	3.62	$J_{6,7}$ 2.0, $J_{6,5}$ 10.5	
7	3.86	$J_{7,8}$ 4.5, $J_{6,7} < 1.0$		7	n.d.		
8	4.17	n.d.		8	3.94	$J_{8,9a}$ 2.4, $J_{8,9b}$ 6.1, $J_{7,8}$ 8.9	
9a	4.18	$J_{9a,8}$ 4.1		9a	3.90	$J_{8,9a}$ 2.5	12.1
9b	3.70	$J_{9b,8}$ 5.9	11.8	9b	3.65	$J_{8,9b}$ 6.1	12.0

n.d. not determined. * Higher order spin system. Error range ± 0.1 Hz.

Table S2. Intra-residue NOE restraining values (in nm) calculated from NOE observations of Ref. 2. Note that pseudoatom corrections are added to distance restraints involving prochiral CH2 or CH3 groups.

Residue a	NOE	r_0 (nm)	Residue b	NOE	r_0 (nm)	Residue c	NOE	r_0 (nm)	Residue d	NOE	r_0 (nm)
H3$_{ae}$-H4	1	0.59	HN-H3$_{ae}$	16	0.59	HN-H3$_{ae}$	35	0.59	HN-H4	53	0.5
H3$_{ae}$-H5	2	0.59	HN-H4	17	0.5	HN-H4	36	0.5	HN-H5	54	0.5
H3$_{ae}$-H6	3	0.59	HN-H5	18	0.5	HN-H5	37	0.5	HN-H6	55	0.5
H6-H11	4	0.6	HN-H6	19	0.5	HN-H6	38	0.5	HN-H7	56	0.5
H7-H8	5	0.5	HN-H7	20	0.5	HN-H7	39	0.5	HN-H11	57	0.6
H7-H11	6	0.6	HN-H8	21	0.5	HN-H8	40	0.5	H3$_{ae}$-H4	58	0.59
H8-H9$_{rs}$	7	0.59	HN-H9$_{rs}$	22	0.59	HN-H9$_{rs}$	41	0.59	H3$_{ae}$-H6	59	0.59
HN-H3$_{ae}$	8	0.59	HN-H11	23	0.6	HN-H11	42	0.6	H4-H5	60	0.5
HN-H4	9	0.5	H3$_{ae}$-H4	24	0.59	H3$_{ae}$-H4	43	0.59	H5-H6	61	0.5
HN-H5	10	0.5	H3$_{ae}$-H5	25	0.59	H3$_{ae}$-H5	44	0.59	H6-H7	62	0.5
HN-H6	11	0.5	H4-H11	26	0.6	H3$_{ae}$-H6	45	0.59	H8-H9$_{rs}$	63	0.59
HN-H7	12	0.5	H5-H6	27	0.5	H4-H5	46	0.5			
HN-H8	13	0.5	H6-H7	28	0.5	H5-H6	47	0.5			
HN-H9$_{rs}$	14	0.59	H6-H8	29	0.5	H6-H8	48	0.5			
HN-H11	15	0.6	H6-H11	30	0.6	H6-H11	49	0.6			
			H7-H8	31	0.5	H7-H8	50	0.5			
			H7-H9$_{rs}$	32	0.59	H7-H11	51	0.6			
			H7-H11	33	0.6	H9$_{rs}$-H11	52	0.69			
			H8-H9$_{rs}$	34	0.59						

Table S3. Inter-residue NOE restraining values (in nm) calculated from NOE observations of Ref. 2. Note that pseudoatom corrections are added to distance restraints involving prochiral CH2 or CH3 groups.

Residue a-b	NOE	r_0 (nm)	Residue b-c	NOE	r_0 (nm)	Residue c-d	NOE	r_0 (nm)
H4-H3$_{ae}$	64	0.59	H6-H3$_{ae}$	73	0.59	H7-H3$_{ae}$	78	0.59
H5-H3$_{ae}$	65	0.59	H7-H3$_{ae}$	74	0.59	H8-H3$_{ae}$	79	0.59
H6-H3$_{ae}$	66	0.59	H8-H3$_{ae}$	75	0.59	H9$_{rs}$-H3$_{ae}$	80	0.68
H7-H3$_{ae}$	67	0.59	H9$_{rs}$-H3$_{ae}$	76	0.68	H9$_{rs}$-H5	81	0.59
H8-H3$_{ae}$	68	0.59	H8-H4	77	0.5	HN-H3$_{ae}$	82	0.59
H9$_{rs}$-H3$_{ae}$	69	0.68						
H9$_{rs}$-H4	70	0.59						
HN-H3$_{ae}$	71	0.59						
H5-H3$_{ae}$	72	0.59						

Table S4. Cremer-Pople parameters for the idealized ring conformations[a]

Conformation	θ [°]	ϕ [°]	Q [Å]
$^{5}C_{2}$	0	0-360	0.57
$^{2}C_{5}$	180	0-360	0.57
$B_{4,O}$	90	0	0.76
$B_{3,6}$	90	120	0.76
$B_{2,5}$	90	240	0.76
$^{4,O}B$	90	180	0.76
$^{3,6}B$	90	300	0.76
$^{2,5}B$	90	60	0.76
$^{2}S_{4}$	90	30	0.62
$^{2}S_{6}$	90	90	0.62
$^{4}S_{6}$	90	150	0.62
$^{4}S_{2}$	90	210	0.62
$^{6}S_{2}$	90	270	0.62
$^{6}S_{4}$	90	330	0.62

[a] Parameters are taken from Ref. 3 where other ring puckering coordinate definitions can be found as well. See Figure S5 for the graphical representation of the coordinates.

1. Nagae, M.; Ikeda, A.; Hane, M.; Hanashima, S.; Kitajima, K.; Sato, C.; Yamaguchi, Y. Crystal Structure of Anti-polysialic Acid Antibody Single Chain Fv Fragment Complexed with Octasialic Acid. *J. Biol. Chem.* **2013**, *288*, 33784–33796.
2. Battistel, M.D.; Shangold, M.; Trinh, L.; Shiloach, J.; Freedberg, D.I. Evidence for helical structure in a tetramer of α2-8 sialic acid: Unveiling a structural antigen. *J. Am. Chem. Soc.* **2012**, *134*, 10717–10720.
3. Hill, A.D.; Reilly, P.J. Puckering coordinates of monocyclic rings by triangular decomposition. *J. Chem. Inf. Model.* **2007**, *47*, 1031–1035.
